# Ask the experts webinar: serious skin infections

**DOI:** 10.1093/jacamr/dlz012

**Published:** 2019-04-12

## Abstract

**Graphical Abstract:**
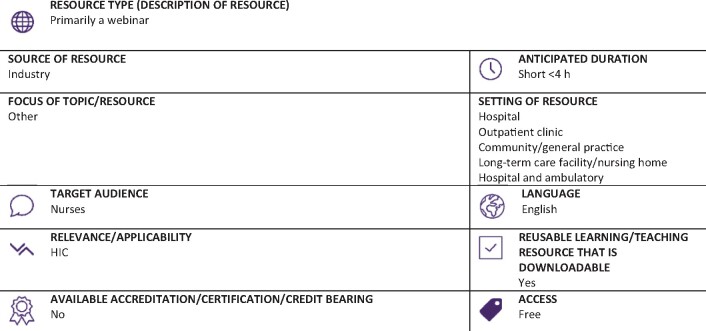

**Resource web link:** **https://rcni.com/primary-health-care/newsroom/sponsored/ask-experts-webinar-serious-skin-infections-141721** (Full classification scheme available at: http://bsac.org.uk/wp-content/uploads/2019/03/Educational-resource-review-classification-scheme.pdf)

**WHO region and country (World Bank):** Europe, UK (HIC)

## Peer review commentary

This resource is an industry-prepared (Correvio) 13 min webinar about the treatment and management of serious skin infections.

The webinar comprises Richard Hatchett, a nurse tutor and senior nurse editor at RCNi, Sharon Falconer, a specialist outpatient parenteral antimicrobial therapy (OPAT) nurse from Aberdeen, and Malcolm Bain from the medical department at Correvio.

Richard leads the discussion by asking questions received from nurses in practice. This is an informal, conversation-based resource; there is little text on screen and answers are given based on experience rather than scientific evidence.

This resource is useful as an introduction to the challenges associated with treating different patient groups and how nursing practice is adapting to these challenges. No prior knowledge is required to benefit from this resource. A range of questions are covered, such as how to treat patients with dementia who have serious skin infections and live in a nursing home, how to determine the origin of a rash (allergy or infection), and how to provide continuity of care for patients that are not admitted to hospital.

Dalbavancin is specifically mentioned, as are journals available on the RCNi website.

This resource is likely to be of use in settings that have a similar healthcare provision to that in the UK. It could be of use to those wanting an insight into nursing practices in the UK.

